# A case of severe bilateral corneal ulcer associated with sintilimab therapy: case report and literature review

**DOI:** 10.3389/fphar.2026.1857433

**Published:** 2026-07-14

**Authors:** Lei Wang, Chun Zhang, Shancheng Si, Junyi Wang, Xuan Liu

**Affiliations:** Department of Ophthalmology, Beijing Tsinghua ChangGung Hospital, School of Clinical Medicine, Tsinghua Medicine, Tsinghua University, Beijing, China

**Keywords:** case report, corneal ulcer, immune-related adverse event, PD-1 inhibitor, sintilimab

## Abstract

**Background:**

Immune checkpoint inhibitors, including PD-1 inhibitors such as sintilimab, have revolutionized cancer treatment but may induce immune-related adverse events (irAEs). Ocular irAEs, particularly severe corneal complications, are increasingly recognized but remain underreported, especially with sintilimab.

**Methods:**

We report a case of a patient in their 60 s with gastric adenocarcinoma who developed bilateral severe corneal ulcers following sintilimab therapy. A comprehensive review of literature on PD-1/PD-L1 inhibitor-associated corneal lesions was conducted to contextualize this rare complication.

**Results:**

The patient presented with progressive ocular symptoms 3 weeks after initiating sintilimab. Despite topical anti-inflammatory and antimicrobial treatment, bilateral corneal ulcers progressed, culminating in corneal perforation of the right eye. Conservative medical management led to gradual healing and anterior chamber reformation. Literature review identified few similar cases, highlighting the rarity and severity of sintilimab-associated corneal toxicity.

**Conclusion:**

This case underscores the potential for severe, vision-threatening corneal complications following PD-1 inhibitor therapy. Early recognition, interdisciplinary collaboration, and timely intervention are critical to mitigate ocular morbidity. Ophthalmologists and oncologists should maintain high vigilance for ocular irAEs in patients receiving immune checkpoint inhibitors.

## Case presentation

A patient in their 60 s was admitted in late August 2025, due to “bilateral visual decline accompanied by eye redness, eye pain, and foreign body sensation for over 6 months, worsening in the past 2 days”.

The patient was previously diagnosed with “gastric malignancy (adenocarcinoma)” in December 2024 and received immunotherapy with sintilimab (Tyvyt®). Immunotherapy was discontinued in March 2025, and the patient underwent surgical treatment for gastric malignancy. The patient denies a history of hypertension or diabetes.

The patient’s course has been prolonged and recurrent, which can generally be divided into the following three stages.

### Stage 1: initiation of immunotherapy and onset of ocular symptoms

Due to gastric malignancy, the patient began immunotherapy with sintilimab on 10 January 2025 (dose: 200 mg intravenous infusion). Approximately 3 weeks later (1 February 2025), the patient developed symptoms such as bilateral eye redness and foreign body sensation. An ophthalmology outpatient visit revealed bilateral conjunctival hyperemia. Treatment included sodium hyaluronate eye drops for lubrication, prednisolone acetate eye drops, and tobramycin-dexamethasone eye ointment for anti-inflammatory purposes. Symptoms improved after treatment. The patient received another dose of sintilimab on 28 February 2025 (dose: 200 mg intravenous infusion). Over the following 4 months, the patient attended regular outpatient follow-ups, and her condition remained stable.

### Stage 2: prolonged and recurrent disease course

**FIGURE 1:**
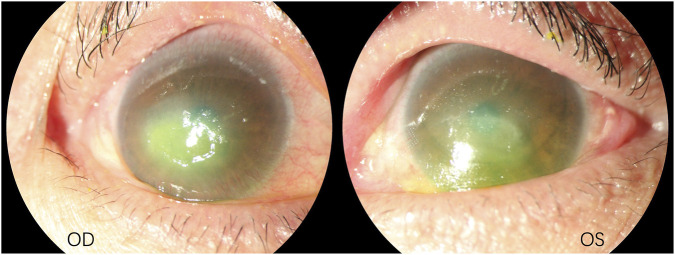
Bilateral corneal ulcers with stromal edema and infiltration (OD: right eye; OS: left eye).

On 12 June 2025, approximately 4 months after the second cycle of Sintilimab, the patient’s ocular symptoms worsened. An outpatient examination revealed extensive corneal epithelial defects (8 mm × 8 mm) and corneal stromal edema in both eyes. The attending physician suspected drug-induced corneal toxicity and discontinued all eye medications except for sodium hyaluronate drops. On 10 July 2025, the patient presented again with worsened symptoms. Examination revealed bilateral corneal ulcers with relatively clean ulcer beds, stromal edema, and hypopyon, leading to a suspicion of endophthalmitis. Microbiological evaluation of corneal scrapings was negative for bacteria, fungi, and *Acanthamoeba* on both stains and cultures. In addition, B-scan ultrasonography identified no evidence of posterior segment involvement (e.g., vitritis, retinitis, choroiditis, or intraocular mass). Despite these findings, empiric anti-infective treatment was initiated; however, it yielded no clinical improvement. The following day, she was seen in the cornea specialty clinic. The treatment regimen was adjusted to include gatifloxacin for infection prophylaxis, frequent application of sodium hyaluronate drops for lubrication, low-concentration dexamethasone eye drops for inflammation control, and therapeutic bandage contact lenses for both eyes. Between July 11 and August 25, the corneal ulcer areas gradually decreased in size, and the hypopyon gradually resolved, indicating a period of stabilization (Figure 1). A Schirmer’s test performed during this period showed a result of 0 mm. Consequently, bilateral punctual occlusion was performed. The corneal ulcers in both eyes subsequently showed signs of gradual healing.

### Stage 3: acute exacerbation

**FIGURE 2:**
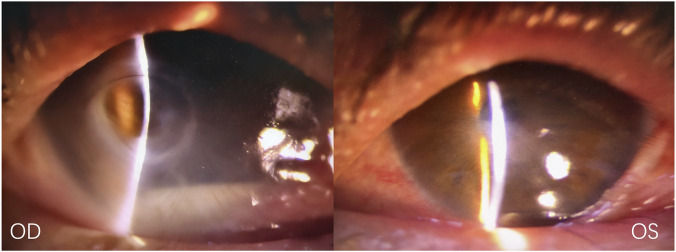
Right eye showing mixed conjunctival injection, large corneal ulcer area with stromal edema and infiltration, corneal thinning, descemetocele, and hypopyon. Left eye showing corneal ulcer with stromal edema and infiltration (OD: right eye; OS: left eye).

On August 27, the patient’s symptoms significantly worsened. Examination revealed progression of the corneal ulcer in the right eye, with descemetocele formation and hypopyon (Figure 2). She was subsequently hospitalized.

### Admission examination findings

Visual Acuity: Right eye: Counting fingers at 30 cm; Left eye: 0.1.

**FIGURE 3:**
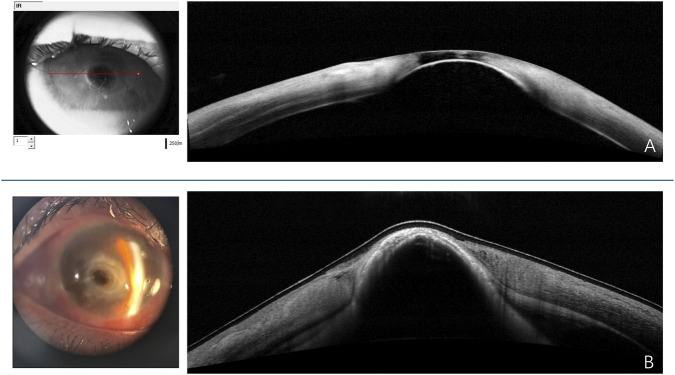
**(A)** Anterior Segment OCT of the right eye, revealing diffuse corneal stromal edema, localized extreme thinning in the paracentral region, and descemetocele formation. **(B)** Rupture of descemetocoele and corneal perforation in the right eye, shallow anterior chamber, with iris tissue plugging the fistula. And the anterior segment OCT, showing corneal perforation in the right eye with iris tissue plugging the fistula.

Both eyes showed symblepharon and mixed conjunctival injection. Large corneal ulcers were observed bilaterally, more severe in the right eye, accompanied by corneal stromal edema and infiltration, corneal thinning, descemetocele, and hypopyon (Figure 2). Anterior Segment OCT of the right eye revealed diffuse corneal stromal edema, with localized extreme thinning in the paracentral region and descemetocele formation (Figure 3A).

### Treatment course

On the second day of hospitalization, the patient’s right eye experienced rupture of the descemetocoele, resulting in corneal perforation (Figure 3B).

**FIGURE 4:**
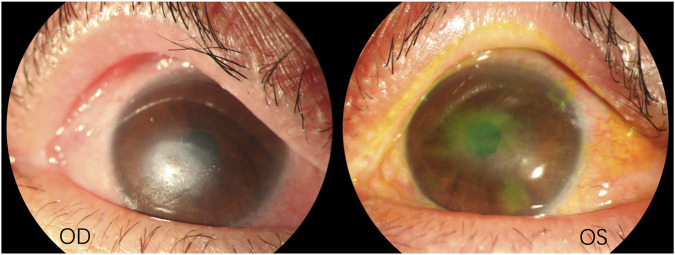
Right eye showing healed corneal ulcer with reformed anterior chamber and residual corneal leukoma. Left eye showing improved corneal ulcer and reduced stromal edema compared to previous status.

The topical medication regimen was adjusted for the right eye to include gatifloxacin eye drops, tobramycin eye drops, levofloxacin hydrochloride ophthalmic gel for infection prophylaxis, and deproteinized calf blood extract eye gel to promote corneal epithelial repair. Following conservative medical treatment, the corneal ulcer in the right eye gradually healed, and the anterior chamber reformed (Figure 4).

## Discussion

Tumor immunotherapy has become a revolutionary breakthrough in cancer treatment, with strategies including cytokine therapy, cancer vaccines, cell therapy, immune checkpoint inhibitors (ICIs), and specific antibodies. Currently, widely used ICIs in clinical practice include programmed death protein-1 (PD-1) inhibitors, programmed death-ligand 1 (PD-L1) inhibitors, and cytotoxic T-lymphocyte-associated antigen-4 (CTLA-4) inhibitors (Song et al., 2025). The PD-1/PD-L1 pathway plays a crucial role in tumor immune evasion—the binding of PD-L1 expressed on tumor cells and other immune cells within the tumor microenvironment to PD-1 on activated T cells initiates inhibitory signaling cascades within T cells, leading to T-cell exhaustion or dysfunction, thereby weakening their cytotoxic effects against tumor cells (Ai et al., 2020; Aden et al., 2025). Sintilimab, a fully human IgG4 monoclonal antibody, specifically binds to the PD-1 receptor on T cells, blocking this immunosuppressive pathway while enhancing the cytotoxicity of tumor-infiltrating lymphocytes against tumor cells (Hoy, 2019). This drug was first approved in China in 2018 for treating Hodgkin lymphoma, non-small cell lung cancer (NSCLC), hepatocellular carcinoma, and malignancies of the esophagus and gastrointestinal tract (Xu et al., 2023).

However, while ICIs activate anti-tumor immunity, they may also disrupt T-cell tolerance to self-antigens, potentially triggering immune-related adverse events (irAEs). Common irAEs can affect the skin, gastrointestinal tract, endocrine glands, liver, and lungs (Ladjevardi et al., 2024; Yan et al., 2025; Zan et al., 2025). Furthermore, ICIs can disrupt the immune privilege of immune-privileged sites, such as ocular tissues, leading to ocular inflammation. Reports of ocular irAEs were relatively rare initially, with reported incidence rates of approximately 0.5%–3%. However, with the increased clinical use of ICIs and growing awareness of ocular irAEs, their reported incidence has gradually increased. Some studies suggest the actual incidence may be as high as 10% (Baughman et al., 2017; Young et al., 2021; Shahzad et al., 2021; Mazharuddin et al., 2022).

Ocular irAEs can involve various ocular structures and adnexa, including uveitis, neuropathies, periocular inflammation, corneal and ocular surface inflammation, retinitis, and paraneoplastic syndromes. Among these, uveitis is the most common ocular irAEs, accounting for approximately 50% of cases. Corneal and ocular surface inflammation make up over 10% of cases, with dry eye syndrome being the most frequent ocular surface irAEs. Studies report its incidence ranging from 1.2% to 24.2% (Dalvin et al., 2018; Abdel-Rahman et al., 2017). This wide variation is likely due to the significant differences in the severity of dry eye symptoms. Mild dry eye symptoms can often be alleviated with topical medications and are frequently overlooked, whereas severe dry eye may lead to serious complications such as corneal lesions, ulcers, or perforation. Several cases of keratitis and corneal perforation induced by immune checkpoint inhibitors (ICIs), particularly nivolumab and pembrolizumab, have been documented (Le Fournis et al., 2016; Nguyen et al., 2016; Bitton et al., 2019; Ramaekers et al., 2021; Vanhonsebrouck et al., 2020). To date, only one case report describes dry eye and keratitis associated with Sintilimab (Xiang et al., 2024). This present case, involving bilateral refractory corneal ulcers and right-eye corneal perforation following Sintilimab treatment, appears to be the first such reported instance. Before attributing these findings to sintilimab, however, infectious causes were systematically excluded. Corneal scrapings were negative for bacteria, fungi, and *Acanthamoeba*, and the ulcer bed appeared relatively clean. Empiric anti-infective therapy failed to produce clinical improvement. B-scan ultrasonography showed no evidence of endophthalmitis. Furthermore, the profound aqueous tear deficiency (Schirmer test score of 0 mm bilaterally) is a hallmark of immune checkpoint inhibitor-induced lacrimal gland inflammation. Collectively, these findings confirm an immune-mediated process as the most likely diagnosis.

The mechanism by which ICIs induce DED and keratitis is not fully understood. Studies have found that corneal epithelial cells, stromal cells, and endothelial cells express PD-L1, which is crucial for maintaining the cornea’s immune-privileged status (Sugita et al., 2009; Shen et al., 2007; Wierenga et al., 2019). Inhibition of the PD-1/PD-L1 pathway may disrupt local immune balance, leading to severe dry eye, corneal ulcers, or even perforation. Additionally, in this case, bilateral Schirmer’s test results were 0 mm, suggesting that ICIs may severely impair lacrimal gland function, causing extreme dry eye. Proposed mechanistic hypotheses include: 1) ICIs breaking self-tolerance and activating autoimmune responses, potentially inducing Sjögren’s-like syndrome (Abdel-Wahab and Suarez-Almazor, 2019; Warner et al., 2019); 2) ICIs disrupting corneal immune privilege, promoting T-cell infiltration of the ocular surface and inducing persistent ocular surface inflammation (Nguyen et al., 2016); and 3) ICIs potentially inducing sarcoid-like granulomatous inflammation of the lacrimal glands (Ileana Dumbrava et al., 2018), a pathological change linked to CD8^+^ T lymphocyte infiltration and IL-2 secretion by activated T cells (Ziegenhagen and Müller-Quernheim, 2003).

Literature indicates that the majority of irAEs occur within one to four treatment cycles (Yan et al., 2025). However, they can manifest at any time after initiating ICI therapy. We summarized all reported cases of corneal perforation as irAEs (Table 1), which occurred at intervals ranging from 2 treatment cycles to 5 years after ICI initiation (Nguyen et al., 2016; Ramaekers et al., 2021; Balasubaramaniam et al., 2025; Alkharashi et al., 2023; Schumaier et al., 2024). This suggests that patients remain at risk for irAEs for a considerable period following ICI treatment, underscoring the importance of detailed history-taking. In this case, the patient developed ocular surface inflammation as early as 3 weeks after starting sintilimab therapy. Initial management with topical artificial tears and corticosteroids provided symptomatic relief, yet the condition was not given sufficient attention. The patient continued to receive two additional cycles of sintilimab, after which the disease rapidly progressed and worsened, leading to severe dry eye, persistent corneal epithelial defects, and further complications including corneal ulceration, perforation, and hypopyon.

**TABLE 1:** Summary of reported corneal perforation cases associated with PD-1/PD-L1 inhibitors.

No.	Age/Sex	Cancer diagnosis	PD-1/PD-L1 regimen	Time to symptom onset	Laterality	DOI
1	68M	Non-small cell lung cancer	Pembrolizumab	22 cycles	Bilateral	10.1097/ICO.0000000000002490
2	58M	Metastatic melanoma	Nivolumab	6 cycles	Left eye	10.1097/ICO.0000000000000724
3	53M	Stage IIIB renal cell carcinoma with lung metastasis	Pembrolizumab	5 years	Left eye	10.1080/09,273,948.2025.2456647
4	69M	Hepatocellular carcinoma	Atezolizumab + Bevacizumab	2 cycles	Left eye	10.12659/AJCR.940688
5	65M	Non-small cell lung carcinoma	Durvalumab	2 cycles	Left eye	10.1016/j.ajoc.2024.102074

Effective management of irAEs hinges on early recognition and timely implementation of immunosuppressive or immunomodulatory strategies based on the affected organ and severity of toxicity (Puzanov et al., 2017). Once ocular surface irAEs occur, collaboration between oncology, ophthalmology, and relevant specialties is essential to weigh the risks and benefits and decide whether to temporarily or permanently discontinue ICIs. The core of ocular treatment focuses on controlling inflammation and promoting epithelial repair, primarily using topical steroids, infection prophylaxis, and artificial tears. For severe cases, systemic immunosuppressants or steroids may be necessary. However, in patients with active malignancy, systemic steroids and immunosuppressants carry the risk of suppressing tumor immunity and potentially promoting primary tumor progression. Therefore, close communication with the oncology team is crucial to carefully weigh the risks and benefits.

For patients who develop corneal melting or perforation, various management approaches have been reported. Ramaekers et al. (Balasubaramaniam et al., 2025) initially attempted treatment with tissue adhesive and a bandage contact lens, but the perforation failed to heal, necessitating tectonic penetrating keratoplasty. However, the corneal graft epithelium failed to heal persistently, leading to further graft thinning and melting. Additionally, a corneal perforation occurred in the fellow eye during treatment, ultimately leading to discontinuation of pembrolizumab. Another report of ICI-related corneal perforation also described failure of tissue adhesive and bandage lens treatment, followed by a small-diameter (3 mm) corneal transplant for the perforation site and cessation of Pembrolizumab. The perforation site healed, and the condition remained stable at 3-year follow-up (Balasubaramaniam et al., 2025). Therefore, for such corneal perforations, we recommend attempting conservative treatment first whenever possible. After excluding infection, options like tissue adhesive and bandage contact lenses can be used to achieve rapid wound closure and promote epithelial healing, aiming for formation of an adherent corneal scar. For patients with large perforations or those unresponsive to bandage lens therapy, corneal transplant surgery with the primary goal of repairing the perforation may be considered. Using a graft with the smallest feasible diameter is advisable. To mitigate the risk of persistent postoperative epithelial defects, conjunctival flap covering or tarsorrhaphy can be considered as adjunctive procedures.

We acknowledge that viral PCR testing (e.g., HSV, VZV, CMV) and anterior chamber paracentesis were not performed due to the high risk of corneal perforation; therefore, viral or malignant etiologies cannot be definitively excluded. Nevertheless, the strong temporal relationship, bilateral presentation, profound aqueous tear deficiency (Schirmer 0 mm), and favorable response to corticosteroids collectively support sintilimab-associated irAE as the most likely diagnosis. This case highlights that for patients receiving PD-1/PD-L1 inhibitor therapy, multidisciplinary management is essential. Clinicians should remain vigilant for the possibility of severe ocular irAEs like corneal lesions, striving for early recognition, close follow-up, and timely intervention to minimize the impact of ICIs on visual function.

## Data Availability

The original contributions presented in the study are included in the article/supplementary material, further inquiries can be directed to the corresponding author.
